# Supplementary material for: Are carbon emissions trading and green financial instruments synergistic? -Comprehensive quantitative research based on content analysis
Source: PLoS One. 2024 Mar 7;19(3):e0298601. doi: 10.1371/journal.pone.0298601 (PMC10919601; doi:10.1371/journal.pone.0298601)
Supplement: S1 Data — (ZIP) [file pone.0298601.s001.zip › Data/地级市Z_F工作报告.zip!/╝¬┴╓/╦─╞╜╩╨/└┤╫╘╣1⁄2╓┌║┼í╛┬φ┐╦╩2╛▌═°í┐_2019.docx]

各位代表：

　　现在，我代表四平市人民政府，向大会报告工作，请予审议，并请市政协委员提出意见。

　　一、过去一年工作回顾

　　2018年是四平发展历程中极不平凡的一年。面对经济持续下行、企业经营异常艰难、金融信贷全面收紧、固定资产投资直线下跌、债务风险解决难度加大、各类社会矛盾易发多发等各种难以预料、历史罕见的重大挑战和考验，我们在省委、省政府和市委坚强领导下，在市人大、市政协监督支持下，紧紧团结和依靠全市人民，及时调整工作部署，果断采取一系列措施办法，稳增长、促改革、调结构、惠民生、防风险各项工作在困难中取得一定成效。

　　——全市经济平稳运行。预计全市地区生产总值增长4.5%，工业总产值、增加值分别增长10.3%和5.8%，全市、市区全口径财政收入按可比口径分别增长7.2%、3.2%，社会消费品零售总额增长4.3%，城乡居民人均可支配收入增长6.5%。产业类项目投资比例显著提升，君乐宝年产4万吨酸奶等一批重大项目建成投产，中成套玉米深加工产业园等重大项目成功签约。出台3项保工业运行政策，推动巨元换热器等100户重点企业有效释放产能，四平卷烟厂二类烟产量增加2.3万大箱。实行领导包保和部门联办制度，清理拖欠企业资金4亿元，现代钢铁公司重组进展顺利，10户“双停”企业重启生产，99户企业历史遗留问题得到解决，民营经济主营业务收入增长7%。

　　——产业结构持续调整。落实“三去一降一补”任务，化解商品房库存104万平方米，去玉米库存490万吨，减免企业税费1.5亿元。双辽昊华年产20万吨离子膜烧碱等3个项目列入国家工业企业技改计划，吉春制药、艾斯克等重点企业产品品质和智能化水平持续提升。新增高新技术企业9户、科技“小巨人”企业5户。划定粮食生产功能区和重要农产品保护区670万亩，土地规模化经营比重达到60%，订单农业面积达到35%，新增棚膜面积3.39万亩，建设全程机械化示范区50个，梨树、伊通、双辽同时成为“全国第三批基本实现主要农作物生产全程机械化示范县”。万邦农副产品批发市场等一批服务业项目建成投运，佳乐宝与阿里巴巴成功签约，电商企业突破700家。新建续建亿元以上文化旅游项目8个，全年游客突破500万人次，增长20.4%。

　　——改革开放深入推进。“只跑一次”改革公布事项2781项，占比达到95.3%。一般企业开办审批时间压缩到3天，比国务院要求缩短5.5天。农村土地确权颁证基本完成，农垦改革位列全省综合排名第一。农村集体产权制度改革、教育医疗等领域改革不断深入，70项改革年度任务全部完成。深化金华－四平对口合作，“四平米、香八婺”推介活动成功举办，永康五金直销中心、农发集团年收储10万吨贸易粮等项目相继落地。

　　——生态环境明显改善。向积累数十年的域内河流污染顽疾发起总攻，开工治理项目36个。西湖湿地、中小河流治理工程基本完工。郭家店等5个节点镇污水处理厂完成阶段性验收，市污水处理厂二期稳定运行，南、北河黑臭水体消除比例达到80%。落实“河长制”“湖长制”，开展“退耕还河”和“清河行动”，流转河道保护带土地1.22万公顷，清理河道垃圾和畜禽粪便15.21万立方米，关闭搬迁养殖场119户，处理非法采砂案件39件，三个断面水质达到国家考核要求。水源地一级保护区内居民全部搬迁，安装水源地围栏2.1万米。退耕还林有效巩固，“四边”植树全面展开。秸秆焚烧得到有效控制，露天烧烤全部取缔，大气质量又有提高。黑土地保护深入实施，市区230万吨陈腐垃圾治理取得重大进展，村级生活垃圾处理机制覆盖率达80.9%。办结中央环保督察和省级环保督察交办案件1189件。

　　——民生福祉日益增进。民生工程扎实推进，16所高标准幼儿园和15个自备水源小区改造等项目基本完成。“走遍四平”环境整治活动深入开展，拆除违建2万平方米，解决各类“城市病”1770个，城市管理水平进一步提升。脱贫攻坚成效显著，实施产业扶贫项目67个，完成危房改造1616户，解决8253名贫困人口安全饮水问题，全年实现9092人脱贫、36个贫困村摘帽。民生事业稳步实施，全市新增就业3.6万人、返乡创业农民工1万人，城乡低保标准进一步提高。市区中小学课后服务问题有效解决，“天使行动”和“三个一批”行动计划深入开展。文化“六馆”向群众免费开放，中式八球国际大师赛等体育赛事成功举办。社会大局和谐稳定，圆满完成国家重大会事信访维稳任务，全市刑事案件起数下降28%，生产安全事故起数和死亡人数分别下降13%、15%。

　　一年来，我们持续加强政府自身建设，深入开展干部作风大整顿活动，自觉接受人大法律监督和工作监督，主动接受政协民主监督，151件人大代表建议和171件政协委员提案全部办结。完善政府预算制度，加大审计监督力度，政务公开和权力运行网上公开全面推进。健全应急预案体系，有效应对恶劣天气、群体事件、火险和疫情，全市未发生较大以上突发事件。国家安全、国防动员、人防建设进一步加强，民族团结、宗教和谐的良好局面持续巩固，司法、统计、侨务、外事、广播电视、新闻出版、防震减灾、气象、红十字会和慈善事业等工作也都取得了新成绩。通过我们的不懈努力，全年呈现出一批亮点工作：一是“只跑一次”改革成为全省样板。成功打造全省唯一“无差别全科受理”政务大厅，“警医邮”、24小时自助服务等创新举措得到国务院督查组充分肯定，在全省考核中名列前茅。二是开发区改革释放新活力。出台开发区改革与创新发展实施意见，明确主导产业和重点发展领域，理顺新开区管理体制，红开区、经开区累计移交社会职能34项、划转人员435人，开发区回归经济主战场，聚焦发展、全力招商的局面初步形成。三是“无籍房”问题有效破解。创新实施并联审批、容缺受理、部门联动等举措，7.7万户“无籍房”进入不动产登记程序或完成登记，该做法被国务院作为典型经验全国通报表扬。四是植树造林成效显著。全年造林绿化52万亩，其中辽河流域生态保护带植树9900亩，我市被全国绿化委员会授予“全国绿化模范单位”。五是非法开采矿山全部取缔。关闭取缔118家证照不全矿场，复绿废弃矿场近百公顷，一举解决了存在近50年的矿山无序开采乱象。六是空气质量明显好转。全年PM2.5浓度均值为40微克/立方米，同比下降15%，空气优良天数达到292天，较去年增加16天。七是保护性耕作优势凸显。推广保护性耕作249万亩，有效抵御历史罕见春旱，夺得玉米生产好收成，经验做法在全省示范推广。八是扫黑除恶取得积极战果。打掉黑恶势力团伙36个，破获案件178起，打处203人，扣押、查封、冻结涉案资产13.5亿元，警均排名全省第一。九是群众呼声得到全方位回应。通过建立机制、反馈意见、督导办理、跟踪问效等措施，对人民网地方领导留言板、市长热线、四平贴吧、四平新闻“随手拍”和来信来访等7057件群众反映合理问题进行回复办理，办复率达到95%，赢得广大群众的肯定和认同。

　　各位代表！事非经过不知难。过去的一年，我们在各类矛盾交织、多重挑战并存、各种风险叠加的情况下，取得这些成绩实属不易。这是市委坚强领导的结果，是全市人民团结奋斗的结果。在此，我代表市政府，向人大代表、政协委员、各民主党派、工商联、无党派人士、人民团体和离退休老同志，向全市广大干部群众、政法干警、驻平人民解放军、武警官兵，向驻平中省直单位，向参与和支持四平发展的各界朋友，表示衷心的感谢和崇高的敬意！

　　各位代表，安不忘危、稳不忘忧。对照习近平总书记在深入推进东北振兴座谈会上的重要讲话精神，对照人民群众对美好生活的新期待、新要求，我们还存在许多问题和短板。一是思想观念落后。市场意识不强，市场化机制滞后，政府包打天下的现象还不同程度存在；习惯于倚重扶持、倚重投资、倚重输血，存在较强的路径依赖；小富即安、求稳怕变、因循守旧等思想严重，开拓精神、创新意识不强。二是经济基础不牢。经济总量低、规模小、效益差；财政盘子小，财政收入占GDP比重低，自我造血功能较弱；企业投资意愿不强，市场主体培育与发展滞后；开发区发展主阵地作用发挥不充分，县域经济存在诸多短板；政府债务还本付息压力较大，防范风险工作任务艰巨。三是结构调整任重道远。三次产业结构不均衡，一产品质不优，二产规模偏小，三产层级不高，一二三产融合不够，科技创新对产业优化升级的引领支撑作用不强；基础设施类政府性投资比例过高，产业类、税源类投资占比低。四是经济外向度低。主动融入“一带一路”和东北亚经济圈的意识弱，对外贸易体量小，政府搭台、部门推动、企业主体、社会参与的开放格局尚未形成共识并付诸行动。五是营商环境不优。政府效率、企业效率、市场效率都不高，法治化、便利化程度较低，服务意识不强，仍然存在“卷帘门”“玻璃门”“旋转门”等现象，有“关系”海阔天空、没“关系”寸步难行的问题还没有根本破解；民营经济发展还比较慢、数量还不够多、个头还不够大、体质还不够强。六是民生短板仍然很多。群众关注的地下管廊、文化体育场馆和断头路等“半拉子”工程，受政府债务和金融政策影响处于停滞状态；群众关心的就业、就医、物业、供热、城市管理等难点痛点问题尚未完全解决。七是干部作风亟待转变。“五弊”问题没有彻底根除，一些干部“爱惜羽毛”，为了不出事，不想为、不愿为、不敢为，工作不主动、遇事不担当；务实精神不够，工作标准不高，干事劲头不足，等等。对此，我们将高度重视，认真加以解决。

　　二、今年主要目标及重点工作

　　2019年政府工作总体思路是：高举习近平新时代中国特色社会主义思想伟大旗帜，全面贯彻党的十九大、十九届二中、三中全会和中央经济工作会议精神，认真贯彻习近平总书记对东北振兴工作重要指示精神，深入贯彻省、市经济工作会议以及市委七届五次全会精神，统筹推进“五位一体”总体布局，协调推进“四个全面”战略布局，做到“五个坚持”，打好“三大攻坚战”，抓实“六稳”工作，纵深推进“五市联动”，坚决打赢创新转型、深化改革、生态治理、乡村振兴“四场战役”，加快建设新时代“美丽四平、幸福家园”，以优异成绩迎接新中国成立70周年。

　　全市经济社会发展的主要预期目标是：地区生产总值增长5－6%，规模以上工业增加值增长6%以上，地方级财政收入与上年持平，社会消费品零售总额增长6%左右，单位GDP能耗达到省定标准，城乡居民收入增长与经济增长基本同步。

　　全年重点做好以下八项工作。

　　（一）全力攻坚项目建设，积极扩大有效投资。项目是点，产业是线，经济是面。必须牢牢抓住项目建设这个核心，在扩大投资总量的同时，持续优化投资结构，夯实四平高质量发展基础。狠抓重大项目建设。加强重大项目储备。主动融入全省“一主、六双”产业布局，立足我市产业基础、资源禀赋、区位条件，加快谋划一批带动能力强、税收贡献大的产业项目，力争打造农产品加工、装备制造、现代物流、医药健康等10个百亿级规模产业，全年谋划储备亿元以上重点项目120个以上。精准开展招商引资。举办四平发展大会，开展“七比七赛”活动，实施以商招商、产业招商、委托招商、叩门招商，重点推动比亚迪新能源专用车、北汽汽车零部件、首农农业、华统肉制品等意向性项目签约落地，全年纳入省调度系统到位资金增长12%以上。强力推动项目集中开工。开展“项目建设年”活动，建立“三早”项目清单和责任清单，全力推进中成60万吨燃料乙醇等50个项目“4·18”集中开工，全年新建续建5000万元以上项目130个，其中亿元以上90个、十亿元以上10个，全年固定资产投资增长10%。优化投资结构。加大产业类项目谋划力度，争取国家和省投资、引进企业投资、激活民间投资，加快新天龙蒸馏工段质量升级、精细化工呋喃铵盐搬迁改造等产业项目建设步伐，确保大清鹿苑鹿产业园、益民堂健康产业园、中科院长春应化所科技创新中心等项目投产达效，全年力争产业类和税源类项目投资占到70%以上，市场主体投资项目占到70%以上。加强要素保障。建立健全项目推进机制，加快拆迁、审批等重点环节进程，做好土地、资金等要素供给。用好用活12亿元土地收储专项债券，加大土地储备力度，提高土地使用效率。落实重大项目包保责任制、项目服务秘书制，建立工作台账，实行跟踪管理，提高项目核准落户率、开工竣工率和投产达效率。加大对各县（市）区、开发区考核力度，晒出“成绩单”、排出“红黑榜”、分出“三六九等”，对工作不力、排名靠后的进行问责。

　　（二）加快推进产业升级，努力实现高质量发展。坚定不移地推进转方式、调结构，推动“数字四平”建设，加快形成多点支撑、多业并举、多元发展的产业发展格局。深入推进供给侧结构性改革。落实“巩固、增强、提升、畅通”要求，加大破、立、降力度，继续化解过剩产能，有序消化房地产库存，提高金融服务实体经济能力，降低企业生产经营成本，增强微观主体活力，加大基础设施等领域补短板力度。加快发展先进制造业。围绕环长春四辽吉松工业走廊，实施智能化制造和“数字化+传统产业”提升工程，提高农产品加工、装备制造、基础化工、冶金建材“老四样”产业智能化、数字化、现代化水平，大力发展医药健康、新型材料、现代物流、绿色包装“新四样”产业集群，推动新旧动能转换。开展两化融合贯标和制造业服务化试点，重点推进商用车智能转向柱总成、中药数字化提取等项目投产达效。在盘活存量上下足绣花功夫，继续做好向总部争取增加产能、帮助企业开拓市场扩大产能、启动“双停”企业释放产能三篇文章，推动行业平均产能达到60%以上。抓好地产品销售中心和电商销售平台建设，借助“东博会”“广交会”等平台，助推企业开拓市场。推动巨润生物、旭晨胶带等10户企业重启生产，加快实施精细化工、双辽星月等技改项目，工业技改投资增长10%以上。提升发展现代服务业。充分发挥四平区位优势，举全市之力推进汽车、建材、粮食、冷链、石油仓储等物流产业快速发展，加快建设万邦冷链物流、君汇40万吨石油仓储物流等项目，全力打造具有四平特色的现代物流产业集群。加快欧亚商贸综合体、万嘉购物广场、吴中地产城市综合体等重点服务业项目建设，推动传统商贸业业态升级。吸引股份制银行、保险机构和证券机构入驻金融街，努力形成点上集聚、功能集成、效用倍增的现代金融集聚区。大力发展电子商务，深化与阿里巴巴、京东等知名电商平台合作，推动特色农产品线上销售。积极发展红色旅游、满族文化特色旅游和冰雪旅游，探索与横店影视城开展合作，加快“满乡风情·七彩伊通”田园综合体、双辽一马树森林公园等项目建设，高水准办好第四届“满族文化论坛”等系列活动。增强科技创新能力。深化与浙江大学、吉林大学、中科院长春应化所、光机所等高校院所实质性合作，提升换热器国检中心等科技平台市场化运作水平。引导企业加大研发投入，新增科技“小巨人”企业20户、高新技术企业10户。实施“万名大学生留平计划”，发挥好院士工作站和硕博人才作用，让更多人才在四平施展才华、创造财富、实现价值。

（三）扎实抓好“三农”工作，全面推动乡村振兴。大力做好“治河、种树、修路、脱贫、兴业”五篇文章，研究制定专项工作方案，画好路线图，明确施工表，走出具有四平特色的乡村振兴新路。加快推动农业升级。构建现代农业“三大体系”，推广保护性耕作技术，提升农业综合生产能力，新建全程机械化示范区25个，建设集中连片高标准农田34万亩，粮食产量稳定在90亿斤阶段性水平。发展绿色农业和特色农业，创建双辽20万亩杂粮杂豆国家绿色食品原料基地，培育特色产业之乡（村）30个。充分发挥新天龙、天成玉米、伊通温氏、双辽牧原等龙头企业作用，提升农产品精深加工水平，促进农村一二三产融合发展。加快推动农村进步。深化农村集体产权制度改革，探索建立新型农村集体经济运行新机制。提升乡村法治和德治水平，大力培育文明乡风、良好家风、淳朴民风。扎实开展农村人居环境整治三年行动计划，推进农村污水处理、垃圾分类处理、厕所革命和村容村貌提升工程，完成退耕还林、退耕还河、农田防护林任务。全年建设农村公路旧路改造工程502.2公里，通屯硬化路100公里。加快推动农民增收。开展农村股份制试点工作，增加农民财产性收入。培育新型农业经营主体和新型职业农民，成立四平中化现代农业产业联盟，推广MAP示范农场模式，全市规模经营比重稳定在60%以上。推广梨树县先导区棚膜经济发展模式，完善加工、冷藏和销售全产业链条。出台农民工转农技工培训管理办法，打造农村半小时服务圈，农村劳动力转移就业实现35万人。加快县域经济发展。扩大县级管理权限事项，落实配套政策，促进城乡在规划布局、产业发展、公共服务等方面融合发展，支持各地挖掘特色资源、壮大优势产业，打造具有竞争力和支撑力的县域经济体系。加快培育叶赫满族文化旅游、十家堡智慧物流、哈福梅花鹿产业等特色小镇，引领乡村创新发展。

　　（四）不断深化改革开放，充分释放发展活力。今年是改革开放再出发的起步之年，要按照习近平总书记“三个下工夫”“五个有利于”要求，加快补齐体制机制短板，释放改革活力。深化“放管服”改革。打造“只跑一次”改革升级版，推动改革向基层延伸，市县两级事项网上可办率不低于70%，100个高频事项实现“只跑一次”。抓好省政府下放、取消权力落地，动态调整市本级权力清单和责任清单。深化商事制度改革，全面推行“证照分离”，执法部门和执法事项全部纳入“双随机、一公开”范围。加强信用体系建设，营造诚实守信社会环境。加快打造“数字政府”，利用市场资源换投资，运用数字资源拓市场，统筹推进“智慧公安”“智慧医疗”“智慧城市”建设。深化其他重点领域改革。加强与国家和省行政体制改革衔接，高标准完成政府机构改革任务。扎实做好承担行政职能事业单位改革，加强综合行政执法队伍建设。引进战略投资者参与市农联社改制，推动组建四平农商行。深化医药卫生体制改革，抓实分级诊疗和家庭医生签约服务，构建各类医疗机构协同发展的服务体系。稳妥推进考试招生制度改革，加强高考综合改革基础保障条件建设。统筹推进国有林场改革，建立科学林草资源管护体制。毫不动摇支持民营经济发展。组织银企对接，发挥“助保金池”作用，缓解企业融资难、融资贵问题。实施中小企业、个体工商户升级计划，培育100户“个转企”、100户“小升规”。加快企业上市挂牌步伐，进入省级培育库企业达到25户，力争企业科创板上市实现突破。开展为民营企业办实事活动，切实解决企业困难和问题。推动产需对接、工商对接、农超对接，助力企业加快发展。激发和弘扬企业家精神，保护民营企业家人身安全和财产安全。民营经济主营业务收入增长6%。深度推进开发开放。开辟大通道，推进开通“平蒙欧”专列，发展对俄、对中亚、对欧贸易，加快建设四平内陆港和临港工业区，推动四平海关挂牌运行，探索设立保税区，提升外向型经济水平。推进大协作，开展对口合作深化年活动，借鉴金华发展模式，利用金华发展资源，确保义乌市场集团农产品物流等已签约项目落地；推动“四辽铁通”次区域合作取得实质性进展，面向京津冀、长江经济带、粤港澳大湾区，全力招商、寻求合作。建设大平台，发挥开发区项目建设主阵地作用，探索“飞地经济”模式，加大基础设施建设投入，实行灵活干部人事薪酬制度，激发开发区内生动力。

　　（五）持续打好“三大攻坚战”，奋力跨越重大关口。聚焦突出问题，打好重点战役，夯实全面建成小康基础。打好防范化解重大风险攻坚战。全面摸清政府债务底数，分类制定清偿计划和应对预案，严格控制政府投资性项目增长速度，抓好债务存量化解和增量控制。积极争取国家和省级财政支持，努力扩大地方债券发行份额，用好用足国家金融信贷政策，合理调整存量融资期限、品种结构。加快推进城投公司等融资平台的转型经营，提升管理水平，拓宽融资渠道，增加运营收入，提高偿债能力，增强综合实力。治理金融乱象，完善防控机制，加大依法维护金融债权支持力度，推动地方金融机构改制重组和经营转型，及时化解重大金融风险。打好精准脱贫攻坚战。以中央脱贫攻坚专项巡视发现问题整改为契机，坚持问题导向，强化资金投入，加大产业发展、文化教育、金融服务、基础设施等方面扶贫力度。深入开展兜底保障“四个一批”专项行动，根本解决“两不愁、三保障”问题。研究实施支持“边缘贫困户”发展政策，加大扶贫扶志，巩固脱贫成效。全年实现13396名贫困人口脱贫、35个贫困村退出、双辽市省定贫困县摘帽，完成脱贫攻坚主要任务。打好污染防治攻坚战。全力以赴抓好中央环保督察和省级环保督察反馈问题整改，深入实施辽河流域水污染防治，扎实推进城区排水管网、河岸保护带建设等61个污染治理项目建设，持续推进城市黑臭水体、柴油车等专项整治。对污水处理厂等重点部位实施精准管理，全面提升治污效能。深入开展“造林绿化三年攻坚”行动，落实“四边”植树，全年造林绿化50万亩。巩固扩大矿山整治成果，制定《四平市水源地保护条例》，科学管控保护区内村屯生活垃圾和污水，严格控制保护区周围化肥农药使用。加大“散乱污”企业整治力度，推进燃煤电厂超低排放改造，年底前基本淘汰县级及以上建成区10蒸吨以下燃煤锅炉。抓好秸秆禁烧和综合利用，完善重污染天气应急预案，PM2.5浓度控制在52微克/立方米以内，全年优良天数力争达到300天以上。加快推进黑土地保护项目建设，用好中央财政资金，高标准完成年度试点任务。推广测土配方施肥、精准施肥、高效肥料技术，扩大增施有机肥、玉米秸秆覆盖还田面积，畜禽养殖粪污资源化综合利用率达到80%以上。加强危化品搬迁企业土壤环境管理，彻底解决市区陈腐垃圾问题，提升城乡生活垃圾无害化处理能力。

　　（六）着力加强城市建设，精心打造幸福家园。以创建“全国文明城市”为统领，加强城市建设与管理，推动城市功能、形象、品位全面提升。科学编制发展规划。尊重城市发展规律，转变城市发展方式，提高城市精细化管理水平，大力推进“多规合一”。坚持四梨同城化发展，优化城乡空间格局和产业布局，拉开城市发展框架。完善城市基础设施。把握国家新一轮基础设施建设投资重大机遇，继续推进地下综合管廊后续工程和军民合用机场建设，紫气大路立交桥、东丰路立交桥、西湖街公铁立交桥建成通车，逐步形成“十三横、十二纵”城市道路骨干网络。规范有序运用PPP模式，重点推进图书馆、大剧院、博物馆、滑冰馆、科技馆等项目建设，避免形成“半拉子”工程。结合海绵城市建设，改造南、北河截污干管47公里。加快牙四公路桑树台至四平段、叶马公路二龙湖至叶赫段道路建设，完善城乡道路网络。大力提升城市品质。开展为期一年的土地和房地产市场专项整治，严厉打击违规违法行为，加强土地出让和房地产开发、销售等环节监管，营造良好的房地产市场环境。持续巩固“无籍房”整治成果，坚决遏制新的“无籍房”出现。深化“走遍四平”活动，集中整治餐饮业油烟污染、非法小广告，加快解决架空线缆问题，破解“脏乱差”等城市顽疾。制定实施《四平市物业服务分级收费及规范管理规定》，明确物业企业和业主的权利和义务，规范物业服务与物业费收缴，从根本上解决物业公司与业主之间的各种矛盾和问题。总结推广“华宇幸福汇”社区服务新模式，创建精品小区。打造城市文化名片。系统挖掘我市历史文化、民族文化、红色文化等资源，着力打造具有四平气质、个性和风格的四平文化。通过搭建“四平新风尚”“四平正能量”“四平好人”等载体，讲好四平故事，传递四平声音，提升四平对外形象。扎实开展“文化七进”，推进戏曲进乡村、京剧进校园，丰富公共文化产品供给，支持国有文艺院团改革发展，引导各类文化资源向基层延伸、向社会开放。推进文化与旅游、科技等深度融合，加快培育一批新媒体、文化创意等新兴文化业态，提升文化产业的规模和层次。

　　（七）更加关注百姓需求，用心用力改善民生。坚持把人民的需求侧作为政府工作的供给侧，多做“接地气”“暖民心”、老百姓真正需要的好事实事。扎实推动就业创业。把稳就业摆在突出位置，实施就业创业三年规划，加强高技能人才培养，完善公共就业服务和创业促就业体系。组建创业学院，发挥创业联盟带动作用，推动创业担保贷款改革，优化就业创业环境。城镇新增就业3万人，城镇登记失业率控制在4.5%以内。加快社会事业发展。加强师德师风建设，巩固中小学课后服务工作成果，提升各级各类教育水平。做好迎接国家卫生城市复审工作，推进市结核病医院迁建项目，筹建市卫生应急指挥中心，市疾控中心、结核病防治所年底投入使用。统筹做好“双拥”、退役军人安置和尊重妇女、关爱儿童等工作。提高社会保障能力。落实全民参保登记政策，推动社会保险全覆盖。进一步完善城乡医疗保险政策，实施医疗保险与商业保险合作，不断提高职工医疗保险待遇水平。继续提高城乡低保标准，开展社区智慧养老平台试点建设，农村养老服务大院覆盖率达到70%。加快推进慈善公益事业发展，公益性生态公墓投入使用。

　　顺应群众新期待，满足市民新要求，今年我们将继续办好10件民生实事：一是提高物业管理服务水平，高标准创建20个幸福小区。二是改造城区剩余自备水源供水小区，全部解决市民安全饮水问题。三是启动168兆瓦热水锅炉建设，市区80%以上居民供暖温度达到20℃以上。四是高标准建设6座过街天桥，保障市民安全便利出行。五是发放1000户公租房租赁补贴，减轻低收入家庭住房负担。六是加强全国中小学生研学实践教育营地建设，促进青少年全面发展。七是完成7个社区卫生服务中心标准化建设，提升基层医疗服务能力。八是改造失能失独家庭适老化活动场所200户，便利老年人居家生活。九是解决5.8万农村人口饮水安全问题，切实增强百姓获得感。十是改造农村厕所2.4万户，补齐影响群众生活品质短板。

　　（八）深入开展扫黑除恶，切实维护社会稳定。推进平安建设和法治建设，妥善化解矛盾纠纷，确保全市政治社会大局和谐稳定。提升社会治理水平。纵深开展扫黑除恶专项斗争，重拳打击征地拆迁、信访维稳等领域涉黑涉恶案件，确保百姓安居乐业、社会安定有序。加大公共安全科技信息化投入，推进视频监控点位建设和升级改造。学习“枫桥经验”，构建社会矛盾纠纷多元化解决机制。健全公共法律服务体系，深入推进“七五”普法和“全国禁毒示范城市”创建等工作。全面贯彻落实党和国家民族宗教政策。提升信访工作水平。深入推进信访积案攻坚，推行信访提示单和提级办理制度，推动重大疑难信访问题及时、有效化解。突出源头治理和事前排查化解，发挥基层调解组织作用，完善市县乡村四级信访工作网络，巩固依法治访工作成效。提升公共安全管理水平。压实安全生产责任，加强隐患排查整治，突出抓好控、防、治、管等环节，坚决守住不发生重特大事故的底线。深入细致做好疫情防控工作，全面提升公共卫生和传染病防控水平。加强食品药品安全监管，让人民群众吃得放心、用得安全。

　　三、全面加强政府自身建设

　　完成好全年确定的各项工作任务，政府系统必须牢固树立“四个意识”，坚决做到“两个维护”，自觉接受市委的正确领导、市人大的依法监督和市政协的民主监督，在实干中锻造忠诚，在执行中检验作风，在落实中体现担当，在清廉中永葆本色。

　　（一）重实干。习近平总书记指出，“伟大梦想不是等得来、喊得来的，而是拼出来、干出来的”。我们要把实干的旗帜举得更高，结合“不忘初心、牢记使命”教育活动，守初心、悟初心、践初心，坚决把奉献和拼搏镌刻在发展的旗帜上；把实干的导向树得更牢，从政府班子自身做起，站排头、作表率，发挥“头雁”效应，各个层面都要着力培树一批像丁华一样的实干榜样、担当楷模；把实干的靶心瞄得更准，紧盯今年“八项重点工作”和“十件民生实事”，把任务分解到部门、具体到项目、落实到岗位、量化到人头，不留余地、不遗余力，“跳起来摘桃子”，努力实现更好业绩；把实干的氛围营造得更浓，深入落实《关于进一步激励广大干部新时代新担当新作为的意见》，为实干者壮胆撑腰、鼓劲打气，让干事者有盼头、有奔头、有劲头，坚决不让实干者吃亏、不让担当者流泪。

　　（二）强执行。执行是政府全部工作的生命线，是考验政府效能的决定性因素。我们要抓制度、保执行，完善政府运行机制，修订《政府工作规则》，建立健全重大决策的联席会商、落实推动、信息反馈、第三方咨询论证等制度，严格重大行政决策合法性审查，提高决策质量，建设高效政府；抓管理、强执行，完善岗位绩效考评体系，定性、定量、定人，打破“大锅饭”，不养“白吃饱”，有效破解干好干坏一个样、干与不干一个样的问题；抓公开、促执行，直面考验、直面问题，不回避、不躲闪，主动公开政府重大决策事项和“三公”经费，主动邀请群众列席政府重大会议，既晒“成绩单”，更晒“任务单”，让权力运行得更加自信和阳光。

　　（三）抓落实。政府的一切工作都要做实做细做深，说话不能华而不实，谋划不能粗而不细，办事不能浮而不深。我们要抓牢“一把手”，对占位子、顶帽子、混日子、摆样子的“堂上木偶”绝不姑息迁就；锁定“最后一公里”，把人民群众获得感这个标准牢牢立起来，更加注重基层、注重实效，坚决杜绝虎头蛇尾，把惠民政策送到企业门口、百姓家中；高举“一把戒尺”，严格落实约谈问责机制，实行全程跟踪、督查督导，敢于触及矛盾，敢于较真碰硬，切实把惩戒的板子打下去，把纪律的威严立起来，以强有力的问责倒逼责任落实、工作落地。

　　（四）保廉洁。公生明，廉生威。我们要以永远在路上的韧劲和执着，落实全面从严治党要求，严守政治纪律和政治规矩，严格遵守中央“八项规定”精神，切实履行“两个责任”，全面推进政府系统党风廉政建设，营造良好政治生态；强化依法行政，建设法治政府，自觉做到尊法学法守法用法；强化审计监督，依法依规审计，扎实开展领导干部自然资源资产离任审计，实现干部清正、政府清廉、政治清明。

　　各位代表！山重水复疑无路，柳暗花明又一村。四平正站在化茧成蝶的重要关口，让我们高举中国特色社会主义伟大旗帜，更加紧密地团结在以习近平同志为核心的党中央周围，坚定信心、团结奋进，加快建设新时代“美丽四平、幸福家园”，为迎接新中国成立70周年而努力奋斗！
